# Dissecting the intratumoral microbiome landscape in lung cancer

**DOI:** 10.3389/fimmu.2025.1614731

**Published:** 2025-07-24

**Authors:** Yan Zhao, Zhibo Yang, Dan Wu, Hai Zhao

**Affiliations:** ^1^ Department of Respiratory and Critical Care Medicine, Lanzhou University Second Hospital, Lanzhou, Gansu, China; ^2^ Department of Neurosurgery, 3201 Hospital of Xi’an Jiaotong University Health Science Center, Hanzhong, Shaanxi, China; ^3^ Department of Neurosurgery, The Affiliated Hospital of Qingdao University, Qingdao, Shandong, China

**Keywords:** intratumoral microbiome, lung cancer, tumor microenvironment, microbial interactions, cancer therapy

## Abstract

The discovery of microbial communities residing within tumors has unveiled a new dimension of cancer biology. In lung cancer, the intratumoral microbiome—comprising bacteria, fungi, and viruses—has emerged as a critical modulator of tumorigenesis, immune evasion, therapeutic response, and metastasis. This review comprehensively examines the landscape of the lung tumor microbiota, highlighting its mechanistic roles in shaping the tumor microenvironment, altering host immune responses, and reprogramming of cancer metabolism. We discuss the influence of specific microbial taxa on immunotherapeutic efficacy, including their interplay with immune checkpoints and pro-inflammatory signaling pathways. Moreover, we evaluate current evidence linking microbial signatures for diagnostic and prognostic applications, emphasizing their potential in biomarker discovery and precision oncology. By integrating findings from molecular epidemiology, multi-omics profiling, and preclinical models, this review provides a translational framework for leveraging the tumor-resident microbiota as both a within tumors, we may develop new microbiome-based strategies. These strategies could improve treatment outcomes and help overcome resistance to immunotherapy.

## Introduction

Cancer is a multifactorial disease driven by a combination of genetic factors, environmental influences, and individual lifestyle choices (1). Genetic predisposition and external exposures shape tumor bacterial content, contributing to interpatient variability. This variability significantly enhances the structural and functional diversity of tumors. Consequently, researchers face increasing challenges in designing effective therapeutic strategies (2–4). The tumor microenvironment (TME) serves as the ecological space surrounding the tumor, containing elements such as blood vessels, immune cells, fibroblasts, bone marrow-derived inflammatory cells, signaling molecules, and the extracellular matrix (ECM) (5, 6). This microenvironment is critical for the initiation and progression of cancer.

**Figure 1:**
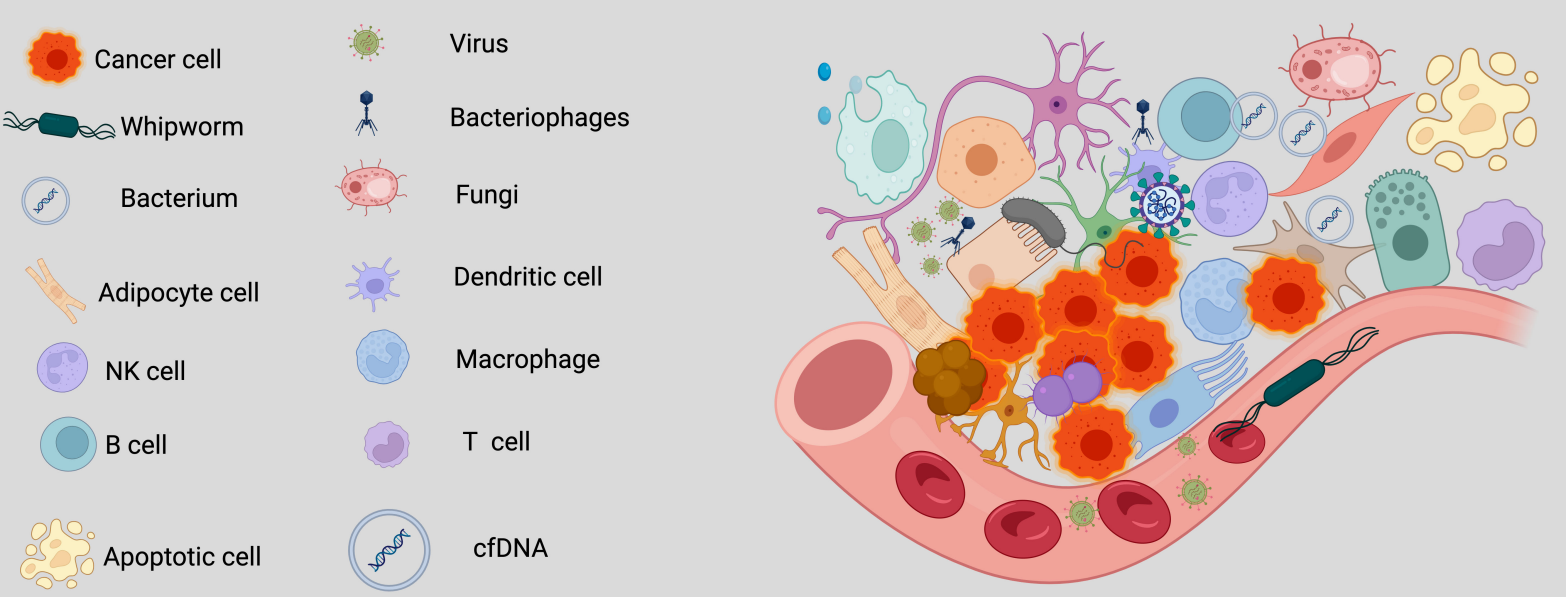
Components of the tumor microbe microenvironment (TMEM). The tumor microbe microenvironment refers to the dynamic and complex interplay between microbial communities (bacteria, viruses, fungi, and other microorganisms) and tumor cells within the tumor niche, alongside host immune, stromal, and vascular components. The evolution from studying the tumor microenvironment to focusing on the tumor microbe microenvironment marks a significant progress in the field of cancer biology. This shift emphasizes the vital influence of microbial populations, including bacteria, viruses, and fungi, within tumors on cancer progression, metastasis, and treatment responses. It highlights the intricate interactions between these microbial communities and other elements of the tumor microenvironment, such as immune and stromal cells, challenging established perceptions of tumor behavior and therapeutic strategies. *Created with* *BioRender.com* .

Over the past decade, scientists have extensively investigated the intricate relationship between cancer and its surrounding environment. Recently, researchers have expanded their focus beyond cancer cells and their immediate surroundings to include a less visible but potentially crucial component—the intratumoral microbiota (7, 8). This shift from focusing on the traditional tumor microenvironment to the tumor microbe microenvironment (TMEM) represents a key advancement in cancer biology (8). In lung cancer, microbial communities, including bacteria, viruses, and fungi, residing within the tumor exert profound influences on tumor growth, metastasis, and therapeutic response (7, 9, 10). This change in focus highlights the essential role of microbial communities in influencing the tumor’s behavior by interacting with immune and stromal cells, thus altering cancer progression and therapeutic outcomes (**Figure 1**).

Additionally, the microbial presence within tumors can modulate immune responses, which may impact the efficacy of treatments such as immunotherapy in lung cancer (11–13). The growing recognition of the tumor microbiome in lung cancer also opens new research avenues, suggesting that targeting the TMEM could lead to novel therapeutic approaches, including microbiome-based therapies and diagnostics. This comprehensive understanding of the tumor microbe microenvironment is transforming cancer research, providing fresh insights and promising pathways for future treatments. Unlike previous reviews that have primarily focused on bacterial associations within tumor tissues, our manuscript uniquely integrates trans-kingdom dynamics—encompassing bacteria, fungi, and viruses—to provide a holistic view of the intratumoral microbiome. We further emphasize mechanistic insights derived from functional experimental models, moving beyond correlative multi-omics studies. Finally, we outline translational pathways—such as standardized clinical microbial profiling, interventional trial frameworks, and regulatory and ethical considerations—that have not been comprehensively addressed in the existing literature. This targeted focus on multi-kingdom interactions, causality, and clinical implementation fills critical knowledge gaps and charts a clear roadmap for future research. This review examines the role of intratumoral microbiota in lung cancer, particularly its influence on disease onset and progression, and explores its potential as a diagnostic and prognostic tool to improve cancer treatment outcomes.

## Historical perspective

The exploration of microorganisms’ role in cancer has evolved over time, with periods of intense interest followed by skepticism. In the late 19th and early 20th centuries, initial observations of bacteria within tumor tissues sparked debates about their possible involvement in cancer development. Pioneers like *William Russell* (1852–1940) coined the term “cancer parasites,” igniting early discussions about the microbial causes of cancer (14). However, due to limited technological capabilities and an incomplete understanding of cancer biology, scientists did not pursue these ideas extensively. A resurgence of interest occurred in the mid-20th century with the discovery of oncogenic viruses, lending more credibility to the idea that microbes could contribute to cancer (15, 16). Research on viruses like *Epstein-Barr* virus and human papillomavirus established strong links between viral infections and certain cancers (17). Despite this progress, attention remained focused on viruses, with less emphasis placed on bacteria and fungi.

It wasn’t until the late 20th and early 21st centuries, with the advent of advanced genomic and molecular techniques, that a more comprehensive understanding emerged. Initiatives such as the H*uman Microbiome Project* have played a key role in unraveling the complex interactions between the human body and its microbial residents (18, 19). These technological advancements enabled researchers to detect and characterize microbial communities within tumor tissues with unprecedented precision, renewing interest in the bacterial and fungal components of the tumor microenvironment, including in lung cancer, where microbial presence has shown microbial presence influences immune responses and immunotherapy outcomes (20).

The 21st century has seen a surge of research in this area, driven by advancements in sequencing technologies. Numerous studies have reported the presence of microbiota within tumors, emphasizing their critical role in the tumor microenvironment and their impact on treatment responses, including immunotherapy in lung cancer (21). The introduction of *next generation sequencing* has accelerated the study of intratumoral microbiota. In 2020, *Poore* et al. explored the diverse intratumoral microbiota across more than 30 cancer types, suggesting a novel diagnostic approach based on microbiota analysis (21). Meanwhile, *Ravid Straussm*an’s team conducted an in-depth analysis of seven tumor microbiomes, revealing their spatial distribution and intracellular localization (22). In 2022, this team uncovered the distribution and synergistic effects of fungi across 35 cancers (23). Concurrently, *Dohlman* et al. analyzed The *Cancer Genome Atlas data*, identifying disease-related fungi in various cancers and investigating the role of fungal DNA (deoxyribonucleic acid) in diagnosis and prognosis (24).

**Figure 2:**
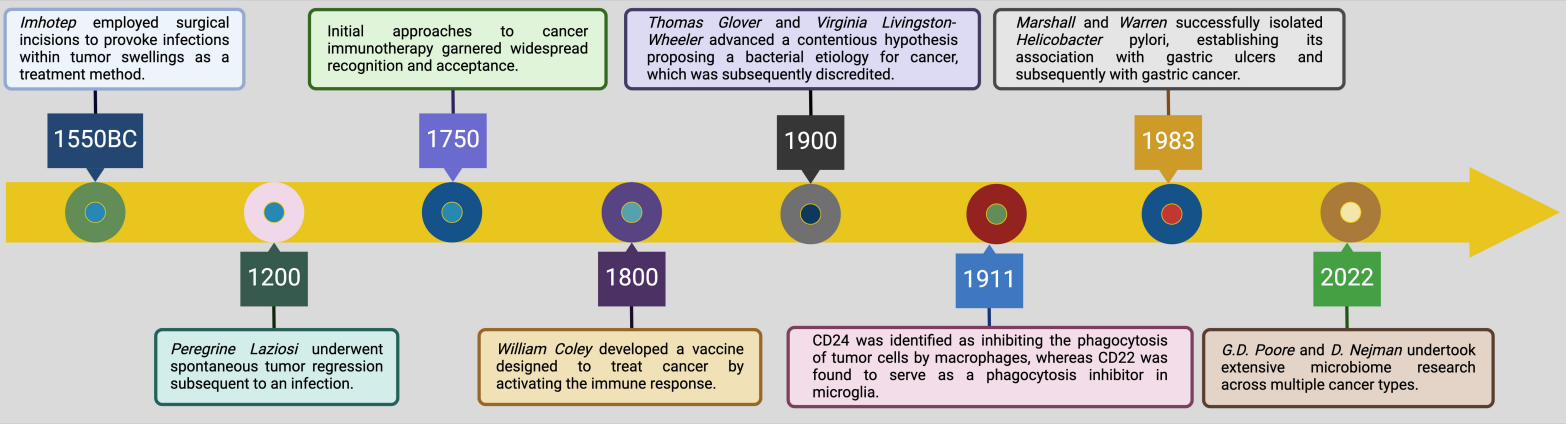
Key research achievements of intratumoral microbiota, tracing back from 1550 BC to the current era. The infographic presents a historical overview of significant research milestones related to the intratumoral microbiota, tracing developments from ancient times to the present. In 1550 BC, *Imhotep* utilized surgical methods to induce infections within tumor swellings as a therapeutic strategy, highlighting the historical acknowledgment of the microbial impact on tumors (28). By 1200, *Peregrine Laziosi* experienced spontaneous tumor regression following an infection, suggesting the potential immune-mediated anti-tumor effects of infections (29). By 1750, initial immunotherapy approaches to cancer began to gain widespread recognition and acceptance, marking the early integration of immunological concepts into cancer treatment (30). In 1800, *William Coley* developed a vaccine to stimulate the immune system against cancer, leveraging the immune response induced by bacterial products to combat tumors (31). In 1900, *Thomas Glover* and *Virginia Livingston-Wheeler* proposed a controversial hypothesis linking bacteria to cancer etiology, though it was later discredited (32). In 1911, discoveries related to CD24 and CD22 began to elucidate their roles in immune evasion within the tumor microenvironment, with CD24 inhibiting the phagocytosis of tumor cells by macrophages and CD22 acting as a phagocytosis inhibitor in microglia (33). The successful isolation of *Helicobacter pylori* in 1983 and its association with gastric ulcers and subsequently with gastric cancer highlighted the definitive role of specific bacteria in cancer development (34). Most recently, in 2022, *G.D. Poore* and *D. Nejman* conducted extensive research on the microbiome across multiple cancer types, further solidifying the critical impact of microbial communities within different tumor environments ^151^. This progression from mere observations to detailed molecular and immunological insights marks significant advancements in the field of cancer biology and treatment. Moreover, the application prospects of CAR-T cell therapy, ferroptosis, cuproptosis, and alkaliptosis in cancer treatment are promising, as they offer novel mechanisms to target tumor cells through immune modulation, metabolic disruption, and induction of non-apoptotic cell death pathways, potentially overcoming resistance to conventional therapies (35–41). *Created with* *BioRender.com* .

Today, the field is entering a new era in which intratumoral bacteria and fungi are no longer viewed as passive bystanders but as active modulators of tumor behavior (25–27). This shift has significant implications for lung cancer immunotherapy, as understanding how the microbiome influences immune modulation can enhance therapeutic strategies. This historical progression, from initial observations to current discoveries, underscores the evolution of scientific thinking and the necessity of interdisciplinary approaches to unravel the complexities of cancer, particularly in the context of immunotherapy (**Figure 2**).

## Associative studies of the microbiome and lung cancer

Advancements in genomic and molecular technologies have dramatically transformed the identification and characterization of intratumoral bacteria. These innovations have enabled researchers to not only detect bacterial presence within tumor tissues but also gain insights into their diversity and potential roles in cancer development. It is estimated that approximately 20% of all cancers globally are influenced by microbial factors (42). In recent years, highly sensitive sequencing technologies have substantially improved microbiome investigations in tissue samples (43). Various studies employing metagenomic approaches have uncovered novel pathogens that are enriched in different cancer types, including lung cancer, compared to adjacent non-tumorous tissues or tissues from healthy individuals (44–46). These findings have allowed researchers to identify microbial DNA signatures in anatomical regions previously believed to be sterile, supporting the concept of cancer-specific microbiomes in organs such as the colon, larynx, and lungs (47–49).

However, while these associative studies provide important insights, they often leave unanswered questions about whether the detected microorganisms are simply coexisting with the tumor or actively contributing to its growth and persistence. The complexity of metagenomic studies and their connection to cancer, particularly lung cancer, has led to ongoing debates (50). Challenges such as differences in microbiome representation in fecal versus biopsy samples, difficulties in accurately assigning genes in metagenomic analyses, and the identification of microbial gene origins in paraffin-embedded tissue samples exemplify some of the obstacles faced in this field.

Moreover, the low bacterial biomass in tumor samples complicates distinguishing true microbial signals from background contamination during DNA extraction. Methodological variations across laboratories in sample extraction, processing, and data analysis can significantly influence results. In particular, contamination from DNA extraction kits (commonly referred to as the “kitome”) has accounted for significant variance in several metagenomic investigations (7, 51). Replicating findings across multiple studies and laboratories is essential for ensuring reliability in this emerging field. Ongoing efforts are actively working toward the harmonization of sequencing protocols and the validation of optimal methodologies. Despite being in its early stages, research shows that a variety of organisms, potentially originating from sources like oral microbiomes, are present in both primary and metastatic lung cancer sites. These microbes may contribute to tumor inflammation through either hematogenous spread or local migration.

This emerging area of research not only challenges conventional views of cancer but also offers fresh perspectives on how intratumoral bacteria could be utilized for diagnostic, prognostic, and therapeutic applications in lung cancer. Continued exploration of these microbial inhabitants holds the potential to add a new dimension to our understanding of cancer biology and treatment.

## Effect of the intratumoral microbiota on human body

**Figure 3:**
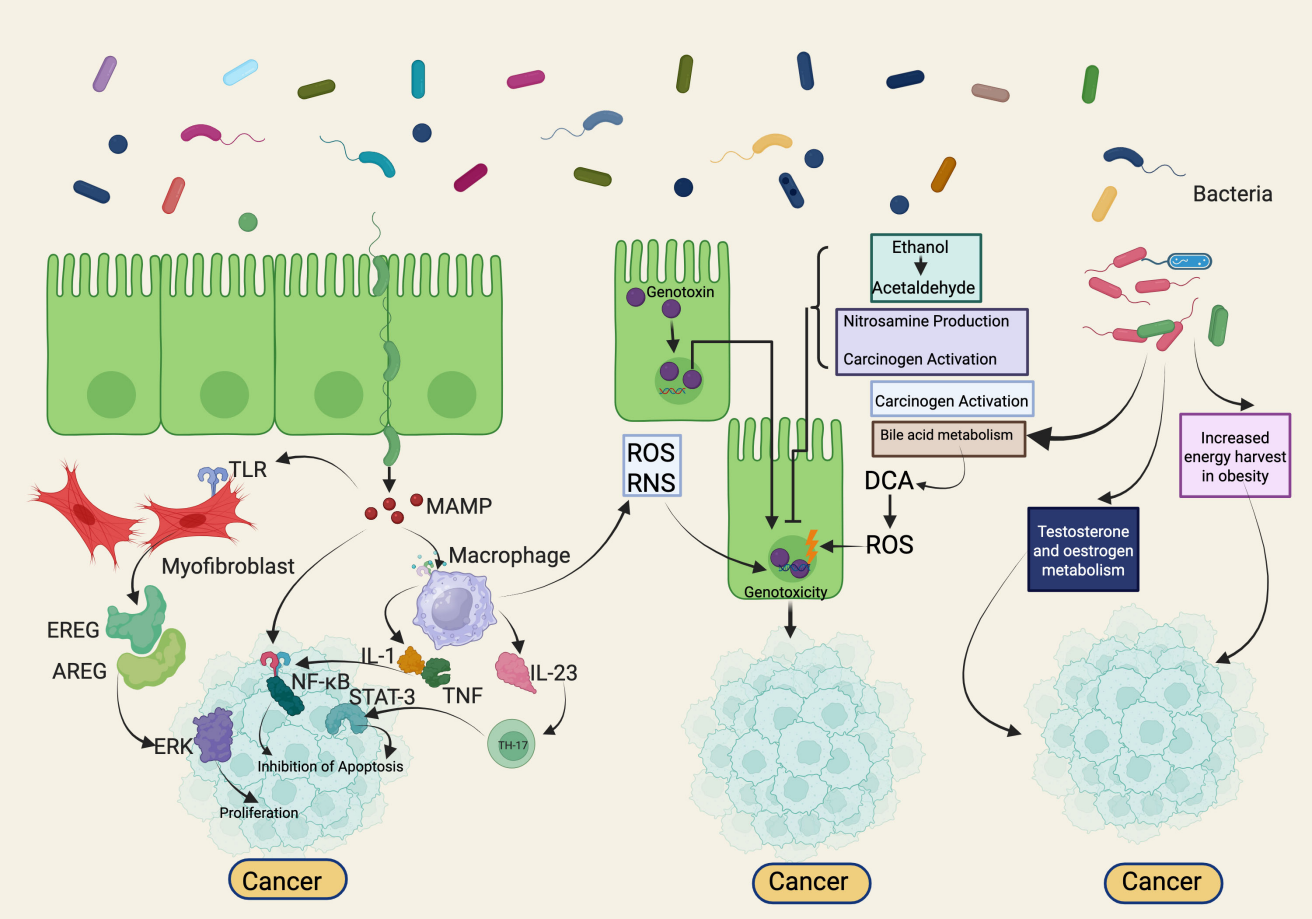
Microbiome-Mediated Mechanisms in Cancer Development: Inflammation, Genotoxicity, and Metabolic Modulation. The bacterial microbiome modulates tumorigenesis through multiple mechanisms. Firstly, disturbances in microbial composition coupled with impaired host immune defenses can enhance tumorigenesis via inflammation and immune modulation, acting both locally at the tumor site and systemically in distant organs. Secondly, certain bacterial toxins, including colibactin and cytolethal distending toxin (CDT), enter host cell nuclei and directly induce genotoxicity, leading to DNA damage within target cells. Lastly, microbiome-driven metabolic activities may activate or produce carcinogenic metabolites such as acetaldehyde, metabolize dietary pro-carcinogens (e.g., nitrosamines), modulate hormone levels (including estrogen, testosterone), alter bile acid profiles, and influence host energy metabolism. Conversely, some microbiota-driven processes may also exert protective anti-tumor effects. *Created with* *BioRender.com* .

The mammalian gut, home to trillions of commensal bacteria, represents one of the most intricate bacterial ecosystems. This diverse microbiota includes not only bacteria but also archaea, protists, fungi, and viruses, with bacteria being the most abundant. These microorganisms play a crucial role in human health by synthesizing vital metabolites, processing nutrients, and producing substances that prevent pathogenic infections while supporting beneficial microbes. They also enhance nutrient absorption and neutralize harmful agents. By interacting with stromal and epithelial cells, the gut microbiota regulates numerous physiological processes. These include preventing pathogen invasion and proliferation, maintaining mucosal immune balance, supporting metabolic regulation, and serving as a protective barrier (52) (**Figure 3**).

In addition to the gut, various body sites—including the skin, nasal passages, respiratory tract, breast ducts, vagina, and gastrointestinal tract—host diverse microbial communities (53–58). Bacteria regularly traverse the gastrointestinal mucosal barrier, entering the enterohepatic circulation, and some may accumulate in tumors, likely due to abnormal tumor vasculature facilitating their residency. Advances in next-generation sequencing have significantly improved our understanding of these microbiotas, addressing the limitations of traditional culture-based methods. The human gut microbiome, which contains as many organisms as human cells, possesses a genome much larger than the human genome itself. Most gut microbiomes have co-evolved with their hosts and contain metabolic pathways not found in the host DNA. The host’s diet, immune system, and epithelial interactions shape the microbiome to fulfill its nutritional needs. *Firmicutes, Bacteroidetes, Proteobacteria*, and *Actinobacteria* dominate the gut microbiota (59–61). Early efforts to classify individuals based on their gut microbiome composition led to the concept of “*enterotypes*,” influenced by factors such as diet, geography, and individual characteristics. However, most microbiome variations appear to follow a continuum that aligns with dietary habits (62).

While the individual microbiome is generally stable, antibiotics can cause significant disturbances, and the impacts of intentional dietary modifications remain insufficiently explored (63). The interactions among less abundant species may be critical for preserving the overall microbiome structure. Studies have linked diet-related bacteria to colon cancer risk, with plant-based diets associated with lower risk. Microbiota-produced short-chain fatty acids, such as acetate, propionate, and butyrate, exhibit anti-inflammatory effects in the colon.

## The characteristics of intratumoral microbiota in lung

The intratumoral microbiota in lung cancer is an emerging area of research that reveals the complex and often overlooked relationship between microorganisms and tumor development (64). Unlike traditional views of tumors as sterile environments, recent studies have shown that lung tumors harbor distinct microbial communities, including bacteria, fungi, and viruses, which can significantly influence tumor progression and therapeutic outcomes (46, 65, 66). Multiple factors shape these microbial communities, including the tumor microenvironment, host immune responses, and underlying comorbidities such as chronic obstructive pulmonary disease (COPD) and pulmonary fibrosis, which frequently occur in lung cancer patients (67, 68). In addition to tumor-intrinsic and immune-mediated factors, the composition of the intratumoral microbiota is heavily influenced by a constellation of host genetic and environmental determinants. Host genetic polymorphisms in immune-regulatory and epithelial barrier function genes can significantly affect microbial colonization and niche selection within tumors (69). Lifestyle-related exposures—including smoking, alcohol consumption, dietary habits, and chronic medication use—further modulate the diversity and functional capacity of the tumor-resident microbiome (70). Moreover, comorbidities such as diabetes and COPD can establish an inflammatory or immunosuppressive milieu, selectively favoring certain microbial taxa (71). Environmental exposures like air pollutants or occupational chemicals may directly perturb microbial ecosystems or indirectly influence them via modulation of host immune responses (72). Therefore, recognizing and adjusting for these confounding variables through stratified study designs, rigorous metadata curation, and multivariate statistical models is essential to accurately elucidate the causal relationships between the intratumoral microbiome and lung cancer pathophysiology. Integrating these host–environment–microbiota interactions into mechanistic frameworks will be critical for the development of reliable microbial biomarkers and for tailoring precision microbiome-targeted therapies.

The microbial composition within lung tumors has been shown to differ markedly from that in adjacent healthy tissue, with bacterial communities exhibiting a higher abundance of species such as *Firmicutes, Proteobacteria*, and *Bacteroidetes* (73). These microbial populations interact with tumor cells, immune cells, and the stromal cells of the tumor microenvironment, influencing key biological processes such as inflammation, immune evasion, and angiogenesis (74–76). For example, certain bacteria may modulate the immune response within the tumor, enhancing the activity of immune cells or promoting tumor-associated inflammation, both of which can either hinder or promote cancer growth. In addition, several microbial taxa have been associated with resistance to chemotherapy and immunotherapy, identifying the intratumoral microbiota as a potential target for novel therapeutic strategies (46, 77, 78).

Furthermore, evidence suggests that the lung cancer microbiota contributes to the metabolic reprogramming of tumor cells by altering nutrient availability and generating metabolites that support cancer cell survival (79). The unique characteristics of the intratumoral microbiome in lung cancer underscore its potential as both a diagnostic marker and a therapeutic target. Understanding the specific microbial signatures of lung tumors could lead to the development of microbiome-based therapies or interventions that enhance treatment efficacy, including the modulation of the microbiota to restore immune function or overcome drug resistance. As research in this field progresses, the role of the intratumoral microbiota in lung cancer continues to reveal itself as a critical component in cancer biology.

## Mechanisms of interaction between intratumoral bacteria and lung cancer

The interaction between intratumoral bacteria and lung cancer is a multifaceted process that involves a complex interplay between microbial communities and the tumor microenvironment. These interactions influence tumor progression, immune evasion, and therapeutic resistance, highlighting the importance of the intratumoral microbiome in cancer biology. Researchers have categorized the mechanisms by which bacteria affect lung cancer into several key pathways.

First, bacteria within the tumor microenvironment can modulate the immune response. Microbes influence the activation and polarization of immune cells, particularly macrophages, dendritic cells, and T lymphocytes, within the tumor microenvironment. For instance, bacterial-derived molecules such as lipopolysaccharides (LPS) or peptidoglycans can trigger the activation of pattern recognition receptors (PRRs) on immune cells, leading to the production of pro-inflammatory cytokines like IL-6 (interleukin-6), TNF-α (tumor necrosis factor-α), and IL-1β (interleukin-1β) (80). This inflammatory milieu can either promote or inhibit tumor growth depending on the nature of the immune response. In some cases, bacteria can stimulate a chronic inflammatory response that fosters tumor progression, while in other instances, bacteria may activate immune pathways that facilitate anti-tumor immunity (81, 82). The specific bacterial species present and their ability to modulate immune checkpoints, such as PD-1/PD-L1 (Programmed cell death protein 1/Programmed death-ligand 1) or CTLA-4 (Cytotoxic T-lymphocyte-associated protein 4), can thus have profound effects on the success of immunotherapies (83, 84).

Second, intratumoral bacteria can influence the metabolic environment of the tumor (85–87). Tumor cells often exhibit altered metabolic pathways, such as increased glycolysis (the *Warburg* effect), to meet their energy demands (88). Bacteria can affect these metabolic shifts by influencing the availability of nutrients, such as amino acids and short-chain fatty acids (SCFAs), which are crucial for tumor cell proliferation and survival (89). For example, certain bacterial species produce SCFAs like butyrate, which can influence histone deacetylation and gene expression, thus altering the epigenetic landscape of tumor cells and promoting tumor growth or immune evasion (90–93).

Third, bacteria within the tumor may contribute to tissue remodeling and the establishment of a permissive microenvironment for cancer progression (94). By secreting extracellular matrix-degrading enzymes or by modulating the activity of stromal cells, such as fibroblasts, bacteria can promote angiogenesis, increase vascular permeability, and facilitate tumor metastasis (95). Additionally, microbial products can influence the activation of signaling pathways such as NF-κB (Nuclear Factor kappa-light-chain-enhancer of activated B cells), MAPK (Mitogen-Activated Protein Kinase), and TGF-β (Transforming Growth Factor-beta), all of which are involved in regulating tumor cell survival, invasion, and metastasis (96).

Lastly, intratumoral bacteria can play a role in mediating therapeutic resistance. The presence of certain bacterial species have been associated with reduced efficacy of treatments like chemotherapy and immunotherapy (97, 98). For example, bacteria that modulate the gut-lung axis or influence the immune microenvironment within the tumor can alter the response to immune checkpoint inhibitors. Microbial-driven changes in tumor metabolism may also result in resistance to chemotherapeutic agents by altering drug uptake or increasing drug efflux (99, 100).

In conclusion, the mechanisms through which intratumoral bacteria interact with lung cancer are diverse and involve the modulation of immune responses, metabolic reprogramming, tissue remodeling, and therapeutic resistance. These findings suggest that targeting the intratumoral microbiome could offer novel approaches to enhance lung cancer treatment, including improving the efficacy of immunotherapies and overcoming resistance to conventional therapies. As our understanding of these microbial mechanisms deepens, the potential for microbiome-based interventions in lung cancer therapy becomes increasingly promising.

### The role of microbiota in lung cancer and tumorigenesis

The human body hosts a variety of microbial communities, especially at barrier surfaces such as the skin. While many of these sites harbor fewer microorganisms compared to the gastrointestinal tract, researchers are beginning to recognize the role of microbial–host cell interactions in tumorigenesis (101). Recent studies have highlighted the role of microbiota in cancer initiation and progression at body sites, such as the lung, which researchers previously believed harbored little to no microbial biomass in the absence of overt infection (42, 102, 103). As a barrier organ exposed to the external environment with each breath, the lung is vulnerable to local inflammation triggered by infections, environmental allergens, pollutants, and cigarette smoke.

Non-small cell lung cancer (NSCLC), the most prevalent form of lung cancer, remains the leading cause of cancer-related deaths globally (104). Understanding the various factors contributing to its carcinogenesis and treatment response is of utmost importance for public health. Additionally, the lung microbiome is emerging as a potential contributor to lung cancer (10). However, the exact role of the lung microbiome in NSCLC is still underexplored, and several studies suggest that viable microbial cells are difficult to isolate from healthy lungs, potentially due to low microbial biomass or technical limitations in detection. Despite this, over half of NSCLC patients have a recent history of bacterial pneumonia or other pulmonary infections (105, 106). Epidemiological studies have demonstrated a strong association between *Chlamydia* pneumoniae infection, chronic inflammation, and lung tumorigenesis.

In NSCLC tissues, the presence of specific microbial taxa has been linked with oncogenic transcriptome profiles, such as the activation of ERK (Extracellular Signal-Regulated Kinase) and PI3K (Phosphoinositide 3-Kinase) signaling pathways (107–109). Further validation came from studies where airway epithelial cells were exposed to bacteria like *Prevotella, Streptococcus*, and *Veillonella* activated PI3K (Phosphatidylinositol 3-Kinase) and AKT (protein Kinase B) signaling both *in vitro* and *in vivo* (110–112). The enrichment of oral bacteria in lung tissue and their role in triggering pathways that contribute to early stages of host cell transformation presents new opportunities for research into lung cancer (113).

**Figure 4:**
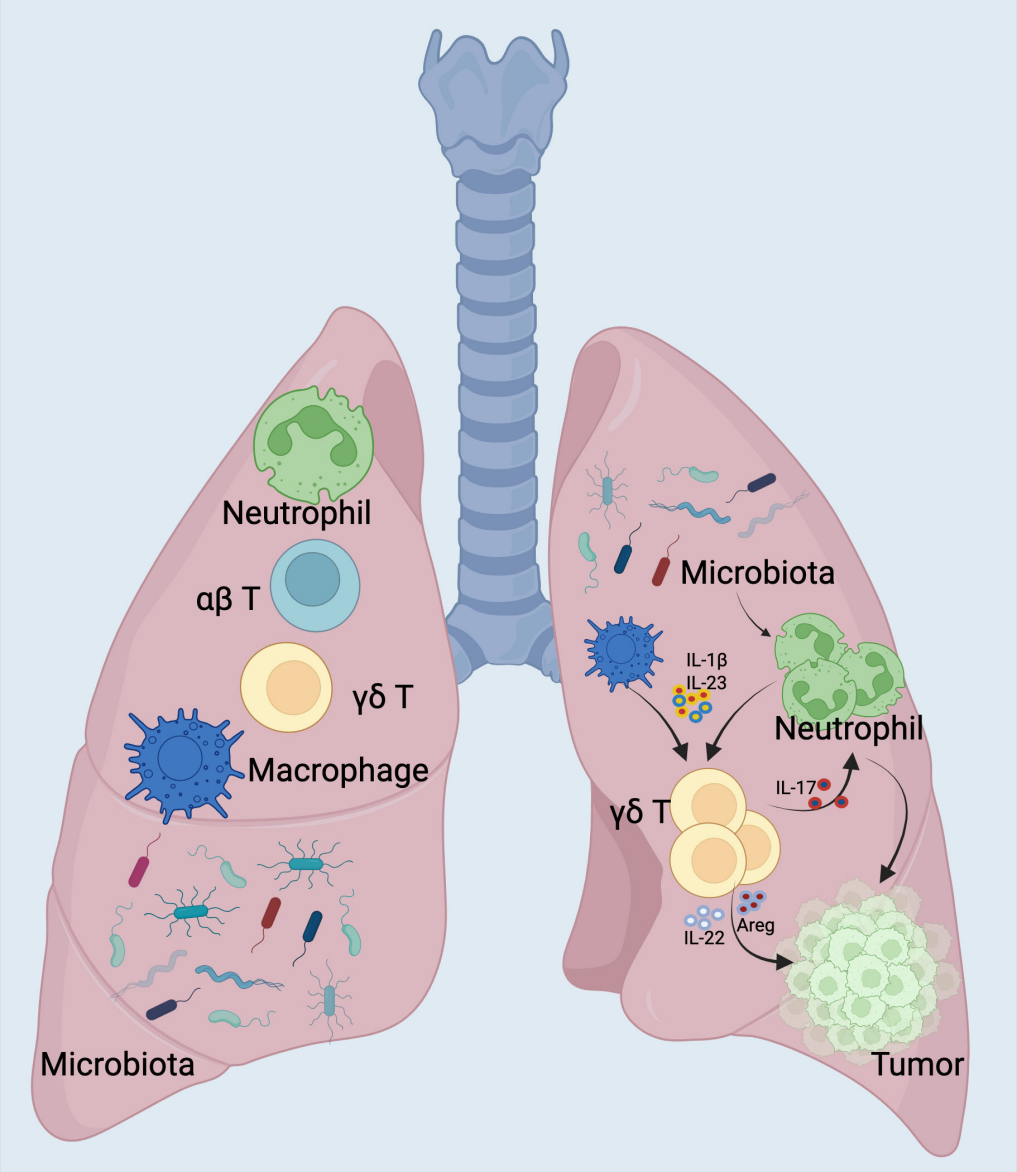
Commensal Microbiota Drive Lung Cancer via γδ T Cell-Mediated Inflammation. The indigenous microbial community incites inflammation linked to lung adenocarcinoma through the stimulation of resident γδ T cells within the lungs. Mice devoid of microbiota or those treated with antibiotics exhibited a marked resistance to the progression of lung cancer triggered by Kras mutation and p53 deficiency (119). On a mechanistic level, resident bacteria prompted myeloid cells to generate IL-1β and IL-23 in a Myd88-reliant manner, which in turn spurred the multiplication and stimulation of Vγ6+Vδ1+ γδ T cells. These cells secreted IL-17 along with additional effector molecules, fostering an environment conducive to inflammation and the proliferation of tumor cells. A definitive connection has been established between the interplay of local microbiota and the immune system in the emergence of lung tumors, pinpointing crucial cellular and molecular agents that could be pivotal in the strategic intervention of lung cancer. *Left* Under normal physiological conditions, lungs maintain a balanced microbiota and immune cell homeostasis, involving alveolar macrophages, neutrophils, and γδ T cells that collectively contribute to tissue immune surveillance and microbial balance. *Right* In lung cancer conditions, local dysbiosis occurs with altered microbiota composition and increased bacterial burden. This microbial dysbiosis activates lung-resident myeloid cells (alveolar macrophages and neutrophils) to produce pro-inflammatory cytokines IL-1β and IL-23. Subsequently, these cytokines stimulate the proliferation and activation of local γδ T cells (predominantly the Vγ6+ subset), promoting their differentiation into IL-17-producing γδ T cells (γδ T17). Activated γδ T17 cells then recruit additional neutrophils and secrete tumor-promoting cytokines (IL-17, IL-22, and amphiregulin [Areg]), contributing to tumor cell proliferation and growth. This positive feedback loop amplifies inflammation and fosters a tumor-supportive microenvironment in the lung, ultimately facilitating lung adenocarcinoma development. *Created with* *BioRender.com* .

In addition to molecular epidemiological studies using human tissues, preclinical models have also been employed to explore the mechanisms by which microbiota may promote lung cancer tumorigenesis (114, 115). *Jin* et al. demonstrated that depleting the microbiota with an antibiotic cocktail in a lung adenocarcinoma mouse model (KP model, which carries a Kras mutation and p53 deletion) significantly reduced lung tumor growth (116, 117). Specifically, they found that a dysbiotic lung microbiota, characterized by an imbalance between symbiotic and pathogenic bacteria, created a pro-inflammatory and pro-tumorigenic tumor environment by stimulating IL-17-producing γδ T cells (118) (**Figure 4**).

**Figure 5:**
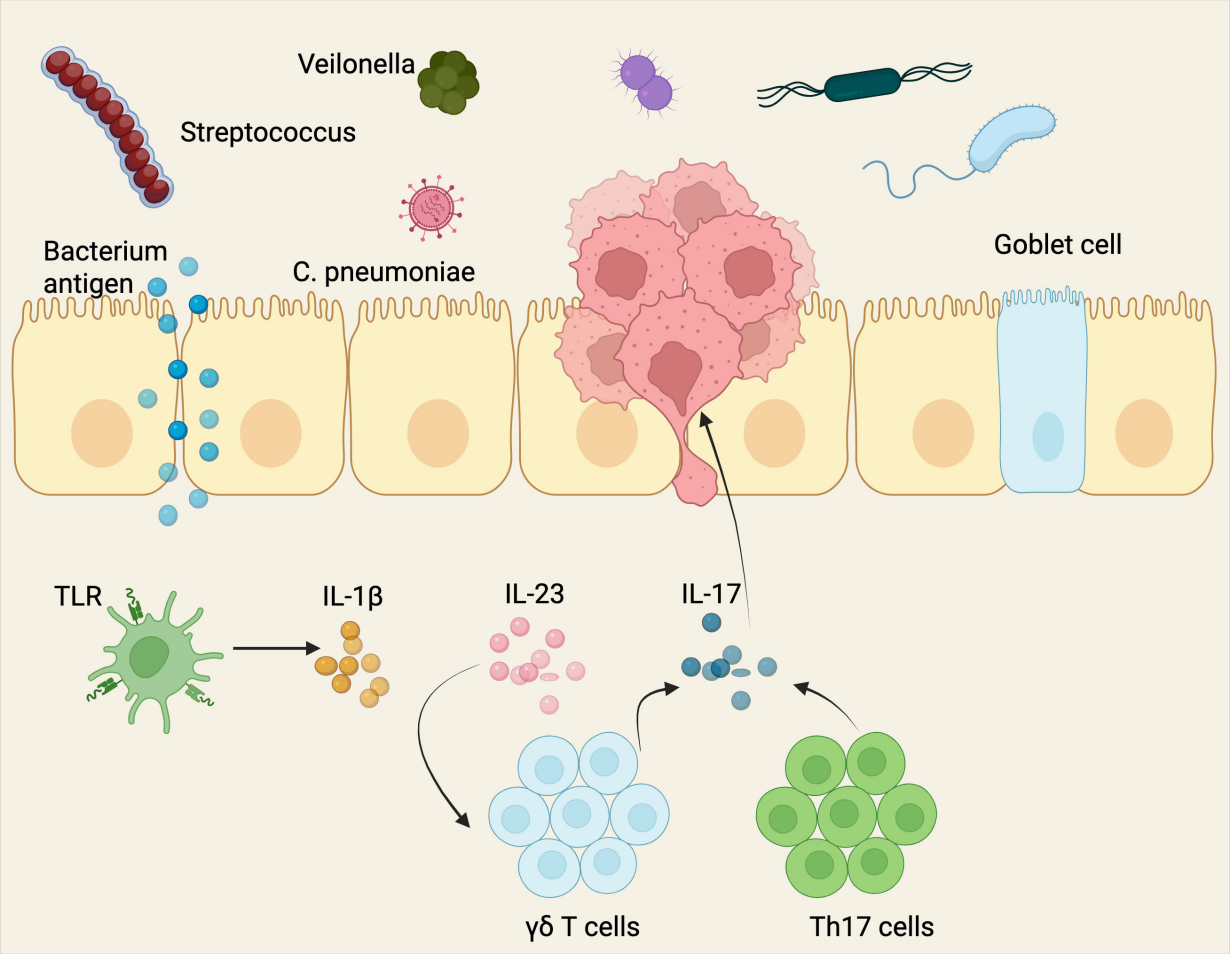
The bacterial microbiome influences NSCLC development via various pathways. The image illustrates the potential role of microbial pathogens and commensal bacteria in promoting NSCLC progression via epithelial barrier disruption and immune modulation. Specifically, microbes such as *Streptococcus, Veilonella*, and *Chlamydia pneumoniae* induce Toll-like receptor (TLR)-mediated signaling, stimulating the secretion of pro-inflammatory cytokines (IL-1β, IL-23), subsequently activating γδ T cells and Th17 cells to release IL-17 (123–125). This inflammatory cytokine cascade leads to epithelial cell changes, immune cell infiltration, and contributes to the tumor-promoting inflammatory microenvironment associated with lung cancer pathogenesis. Mechanistically, these microbiota can directly stimulate the activation of the PI3K–PDPK1 (PDK1)–AKT signaling pathway (96, 126). This microbial-driven signaling promotes oncogenic processes. Additionally, these microbes trigger immune responses characterized by enhanced inflammatory cytokine production, contributing further to tumorigenesis in the lung microenvironment. *Created with* *BioRender.com* .

Researchers observed a marked increase in bacterial load, notably from taxa belonging to the *Herbaspirillum* genus and the *Sphingomonadaceae* family (120, 121). In this study, microbial products (e.g., LPS and peptidoglycan) triggered the activation of TLRs (toll-like receptors), alveolar macrophages and neutrophils, elevated tissue levels of IL-1β and IL-23, and increased the presence of activated lung-resident γδ T cells (122) (**Figure 5**).

**Figure 6:**
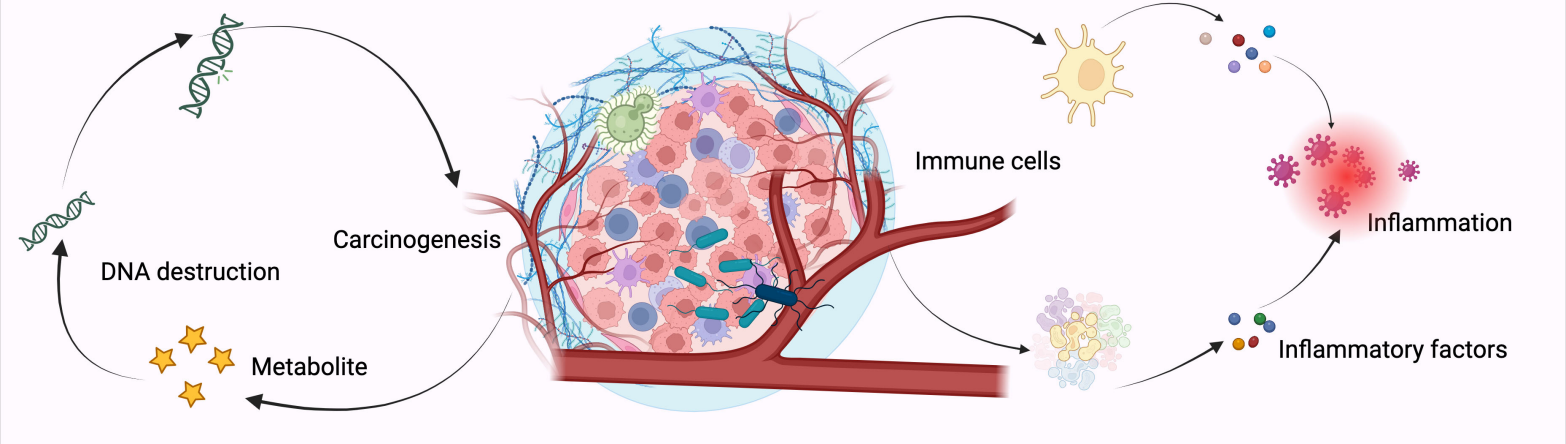
Mechanisms of Intratumoral Bacteria in Lung Cancer Development. The relationship between intratumoral bacteria and cancer cells involves complex mechanisms that are not yet fully elucidated. Current understanding suggests three primary pathways through which these bacteria may influence cancer progression: firstly, the production of genotoxic substances that induce genetic mutations; secondly, the engagement with host cellular signaling pathways implicated in carcinogenesis; and thirdly, the promotion of inflammatory responses and immune system modulation, which can facilitate cancer initiation. These interactions highlight the multifaceted role of intratumoral microbiota in oncogenesis. *Created with* *BioRender.com* .

The detection of microorganisms in organs previously considered sterile or of low biomass, in the absence of infection, may signal the loss of immune-microbial balance, contributing to chronic inflammation, which is a known risk factor for cancer. Persistent dysbiosis during tumor development and progression can alter the immune system, thereby influencing patient outcomes. Chronic inflammation, in particular, is a well-established risk factor for NSCLC, making further mechanistic studies necessary to understand the contribution of microbiota to its initiation and progression (127, 128) (**Figure 6**).

### Mechanisms of intratumoral microbiota in lung cancer metastasis

One of the key mechanisms through which intratumoral bacteria influence lung cancer metastasis is by promoting epithelial-to-mesenchymal transition (EMT), a crucial process for metastasis (91, 129). EMT allows epithelial cells to acquire migratory and invasive characteristics, enabling cancer cells to break away from the primary tumor and invade other tissues. Bacteria such as *Fusobacterium nucleatum* and *Bacteroides fragilis* have been found to enhance the ability of cancer cells to adhere to endothelial cells, facilitating their extravasation into the bloodstream (130, 131). Once in the bloodstream, these cancer cells can spread to distant organs, a fundamental step in metastasis. Additionally, bacterial species present in the lung tumor microenvironment can activate signaling pathways like ERK (extracellular regulated kinases) and PI3K (phosphatidyl-inositol 3-kinase), which further enhance the invasive capabilities of lung cancer cells (132, 133).

Intratumoral microbiota also contribute to the formation of a premetastatic niche (PMN), an environment that prepares distant organs for the successful colonization by metastatic cells (133–135). This occurs through the stimulation of inflammatory pathways that promote immune cell infiltration, blood vessel formation, and the remodeling of the extracellular matrix. For example, bacteria such as *Prevotella* and *Streptococcus* have been shown to trigger the activation of pro-metastatic signaling pathways, which prepare the secondary organs for tumor cell colonization (136). Through these mechanisms, the microbiota actively shapes the tumor microenvironment, making it more conducive to the metastatic spread of cancer cells.

Moreover, intratumoral bacteria have been shown to modulate the immune system, contributing to immune evasion and facilitating metastasis (89). Bacteria can stimulate immune cells such as macrophages and neutrophils, which release pro-inflammatory cytokines that create a microenvironment favorable for tumor growth and metastasis (137, 138). For instance, the presence of *Fusobacterium nucleatum* has been shown to activate the *NF-κB* pathway, which increases the expression of cytokines like IL-6 and TNF-α (139). These inflammatory signals enhance tumor cell survival, angiogenesis, and invasion. Additionally, certain bacteria may modulate the immune checkpoint pathways such as PD-1/PD-L1, which are critical for immune evasion in tumors (140). By promoting the accumulation of immunosuppressive immune cells such as regulatory T cells (Tregs) and myeloid-derived suppressor cells (MDSCs), the microbiota can enhance the tumor’s ability to escape immune surveillance and facilitate metastasis (141).

### Preclinical models and studies on the role of microbiota in lung cancer metastasis

In addition to molecular epidemiological studies using human tissue, preclinical models have provided deeper insights into the mechanisms by which the microbiota contributes to lung cancer metastasis (142–144). For example, studies using mouse models of lung adenocarcinoma, such as the Kras mutation and p53 deletion (KP) model, have shown that depletion of the microbiota using antibiotic cocktails significantly reduces both primary tumor growth and metastasis (131). These findings underscore the important role of the microbiota in promoting metastasis through immune modulation and the creation of a pro-inflammatory environment. The depletion of microbiota in these models also revealed that a dysbiotic microbial community—characterized by an imbalance between beneficial and pathogenic bacteria—can create a tumor-promoting milieu by enhancing the activation of immune cells, such as γδ T cells, that are known to facilitate tumor progression and metastasis (119, 145).

Furthermore, metagenomic analysis of bronchoalveolar lavage fluid in tumor-bearing mice showed increased bacterial diversity, particularly the enrichment of pathogens from the Herbaspirillum genus and *Sphingomonadaceae* family, which correlated with increased metastasis and tumor growth (146–148). The activation of TLRs by bacterial products, such as LPS (Lipopolysaccharide) and peptidoglycan, leads to the activation of alveolar macrophages and neutrophils, which further contribute to a pro-inflammatory microenvironment. This increased immune cell activity results in elevated levels of cytokines such as IL-1β and IL-23, which promote tumor metastasis (149).

## Impact on lung cancer diagnosis and prognosis of intratumoral microbiota

The growing body of evidence surrounding the role of intratumoral microbiota in cancer has opened new avenues for its application as both a diagnostic and prognostic biomarker in lung cancer, particularly NSCLC. The TME is known to be a complex ecosystem that includes not only tumor cells but also stromal cells, immune cells, and microorganisms. The microbial communities residing within the tumor tissue have been shown to influence various aspects of tumor biology, including immune modulation, inflammation, and tumor progression. Understanding the specific microbial signatures associated with lung cancer can therefore provide novel insights into the mechanisms of carcinogenesis and metastasis.

Recent research has demonstrated that the microbial composition within NSCLC tumors significantly correlates with the activation of key oncogenic signaling pathways, including the ERK and PI3K/AKT pathways, which are involved in cell survival, proliferation, and metastasis (46, 110, 150, 151). These microbial signatures have been shown to modulate the host immune response, particularly through the activation of pro-inflammatory cytokines and immune checkpoint molecules, which can promote tumor immune evasion (152). The diversity and specific composition of the intratumoral microbiota have been linked to the presence of certain bacterial species that can either support a pro-tumorigenic microenvironment or enhance antitumor immunity. For instance, certain microbial species, such as *Fusobacterium nucleatum* and *Bacteroides fragilis*, have been implicated in the promotion of cancer cell proliferation, invasion, and metastasis, through mechanisms like EMT and the activation of pro-inflammatory pathways (131, 153).

The ability to profile the intratumoral microbiota in clinical samples provides a promising approach for the early detection of lung cancer. By characterizing microbial communities within tumor biopsies or even non-invasive samples such as sputum, liquid biopsies, or bronchoalveolar lavage fluid, it is possible to identify specific microbial signatures that are uniquely associated with the presence of cancer. These microbial profiles could complement traditional diagnostic methods, potentially offering a more sensitive and specific tool for early cancer detection, particularly in patients with limited clinical symptoms or those at high risk for lung cancer.

In addition to its diagnostic potential, the intratumoral microbiota holds significant promise as a prognostic biomarker in lung cancer (102, 154, 155). The microbial diversity within tumors has been shown to correlate with patient outcomes, including overall survival and response to treatment. For example, certain bacteria present in the TME have been linked to improved responses to immunotherapies, such as immune checkpoint inhibitors, by modulating the immune system to promote a more favorable antitumor immune response. Conversely, dysbiosis, or an imbalance in the microbial community, can result in an immunosuppressive environment, contributing to immune escape and resistance to therapy. As such, microbial profiling could provide critical information on the likelihood of response to specific treatments, including chemotherapy, targeted therapy, and immunotherapy, thereby guiding personalized treatment strategies.

The potential of intratumoral microbiota as a diagnostic and prognostic marker in lung cancer is a promising frontier in cancer research. By leveraging advanced sequencing technologies and bioinformatics approaches to profile the microbial communities within tumors, it is possible to gain a deeper understanding of the complex interplay between microbiota and cancer biology. The identification of specific microbial signatures associated with NSCLC could pave the way for new diagnostic platforms and therapeutic strategies, including microbiome-based interventions aimed at restoring a healthy microbial balance in the TME to enhance treatment efficacy and improve patient outcomes. Continued research in this area will undoubtedly expand the role of the microbiota in lung cancer management, offering a novel layer of precision medicine for patients with lung cancer.

## Therapeutic implications

The discovery of intratumoral microbiota has profound implications for the development of new cancer therapies. By understanding the roles these microorganisms play within tumors, we can explore novel treatment strategies that target or utilize these microbial residents.

The recognition of intratumoral microbiota as a key player in lung cancer progression has profound implications for therapeutic strategies. Recent studies have illuminated the complex interactions between microbial communities and tumor cells, revealing that the microbiota within the TME can modulate tumor biology, immune responses, and treatment outcomes. These findings suggest that targeting the intratumoral microbiota could offer novel therapeutic approaches, complementing existing treatments such as chemotherapy, targeted therapy, and immunotherapy.

One of the most promising therapeutic implications of intratumoral microbiota is the potential to modulate the immune system to enhance the effectiveness of immunotherapy (156). The TME is characterized by a complex interplay between tumor cells, stromal cells, immune cells, and microorganisms. Certain microbial species within the TME have been shown to influence immune cell function, including the activation and polarization of macrophages, dendritic cells, and T lymphocytes. For instance, bacteria such as *Fusobacterium nucleatum* and *Bacteroides fragilis* have been implicated in promoting a pro-inflammatory tumor microenvironment, which can enhance immune evasion and facilitate tumor progression. Conversely, other microbial species have been associated with enhanced antitumor immunity by promoting the activation of cytotoxic T lymphocytes and natural killer (NK) cells (157).

This dynamic between microbial communities and immune responses offers a potential strategy to modulate the TME to favor antitumor immunity. One approach could involve manipulating the microbiota using probiotics or targeted microbial therapies to restore a healthy microbial balance within the TME. This could promote immune activation and improve the efficacy of immune checkpoint inhibitors, such as anti-PD-1 and anti-CTLA-4 therapies. Recent studies have shown that the composition of the gut microbiome can influence the response to immunotherapy, with certain bacterial species enhancing the antitumor immune response. Extending this concept to the lung cancer TME could lead to the development of microbiome-based therapeutic interventions that improve the effectiveness of immunotherapy in lung cancer patients.

Another therapeutic implication involves targeting microbial-driven inflammation, which is a known contributor to tumorigenesis and metastasis. Chronic inflammation, often induced by dysbiosis in the TME, is a key factor in the development and progression of lung cancer. Inflammation promotes tumor cell proliferation, angiogenesis, and metastasis while also contributing to immune suppression. Microbial species that drive chronic inflammation, such as those producing LPS or other pro-inflammatory metabolites, could be targeted to reduce the inflammatory burden in the TME. By using antibiotics or specific antimicrobial agents that deplete pro-inflammatory bacteria, it may be possible to mitigate tumor-promoting inflammation and improve treatment responses. However, the risk of disrupting the overall microbial diversity and causing unintended consequences must be carefully managed, as the TME is home to a variety of microbial species that may exert both beneficial and harmful effects.

Additionally, the microbiota’s influence on cancer cell metabolism and resistance to therapies represents another promising therapeutic target. Intratumoral bacteria have been shown to alter the metabolic landscape of tumors, providing essential nutrients that support tumor cell growth and survival. For example, certain bacteria produce SCFAs, which can influence the tumor’s epigenetic landscape and promote tumor growth (158). By targeting the metabolic pathways influenced by the microbiota, such as those related to SCFA production or nutrient availability, it may be possible to disrupt the metabolic support that tumors rely on, impairing their growth and enhancing the effectiveness of conventional therapies.

Finally, the role of intratumoral microbiota in modulating cancer cell plasticity, stemness, and metastasis suggests that microbial-targeted therapies could reduce the metastatic potential of lung cancer. Bacteria like Bacteroides fragilis have been shown to activate stemness-associated pathways such as the Notch and Wnt/β-catenin pathways, which are crucial for cancer cell survival, proliferation, and metastasis. By targeting these bacterial species or the pathways they influence, it may be possible to reduce cancer cell plasticity, preventing metastasis and improving patient prognosis. Moreover, disrupting the interaction between bacteria and cancer cells could reduce the invasive behavior of tumor cells, impairing their ability to invade surrounding tissues and spread to distant organs (159).

In conclusion, the therapeutic implications of intratumoral microbiota in lung cancer are vast and diverse. By modulating the microbiota within the tumor microenvironment, it may be possible to enhance the effectiveness of immunotherapies, reduce tumor-promoting inflammation, disrupt cancer cell metabolism, and prevent metastasis. These insights open the door to microbiome-based therapies, offering new opportunities to improve treatment outcomes and personalize cancer care for lung cancer patients. However, further research is essential to fully understand the complex relationships between the microbiota and the TME and to develop safe and effective strategies for clinical application.

## Challenges and future directions

While recent advancements have shed light on the potential role of microbes in cancer, including their existence and activities within tumors, this field remains in its early stages and continues to face significant hurdles (156). One of the primary challenges is the presence of microbial communities, including bacteria and fungi, in tumors previously thought to be sterile. This notion remains contentious, and further studies are needed to definitively characterize the microbiota within these tumors, especially in the context of cancers like lung cancer, where microbial involvement in tumorigenesis and progression is still poorly understood. Characterizing these low-biomass tumor microbiomes is complicated by several technical and biological obstacles, such as contamination risks, batch effects, inaccurate data allocation, and imperfections in analytical methods. Moreover, identifying intracellular bacteria and understanding their relationship to specific tumor phenotypes remains a daunting task (156). To advance our understanding, the use of animal models and organoid cultures in the study of bacterial invasion will be pivotal in determining the extent of intratumoral bacterial colonization and its role in cancer progression. These models, particularly in lung cancer research, can provide valuable insights into how bacterial populations interact with cancer cells and how these interactions affect tumor behavior. However, rigorous control measures and analytical methods are essential to eliminate experimental and computational contaminations, as demonstrated by the recent resolution of debates surrounding the existence of a placental microbiome.

**Figure 7:**
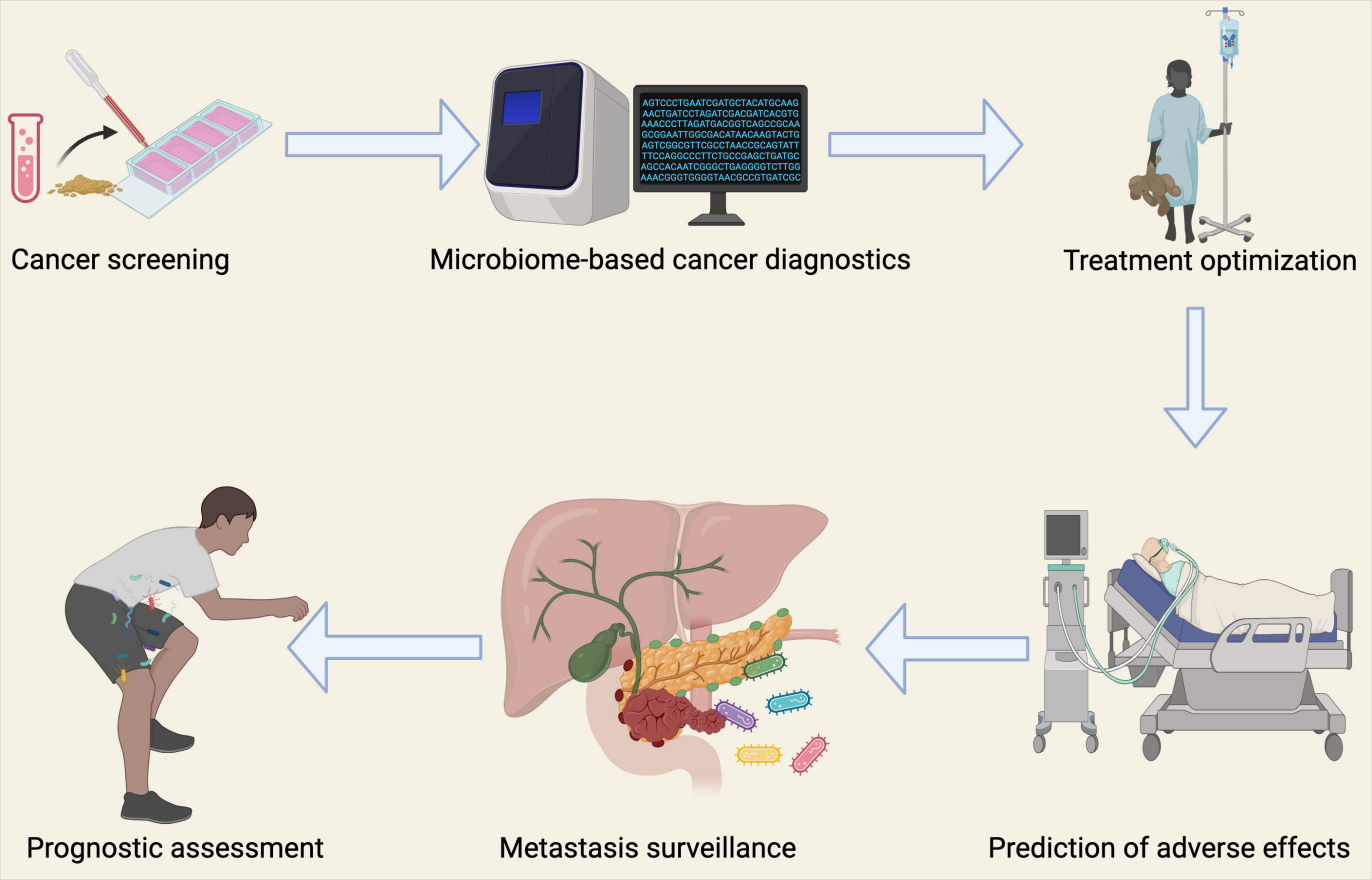
Clinical Implications of the Microbiome in Lung Cancer Diagnostics and Therapy. The bacterial microbiome has important clinical applications in oncology, facilitating lung cancer diagnosis and treatment through multiple strategies. These include detection of circulating microbial DNA in peripheral blood for cancer diagnosis, surveillance of micro-metastatic disease progression, prognostic assessment, tailoring personalized therapeutic regimens, and integration with artificial intelligence approaches to anticipate treatment responses and potential treatment-related adverse events.

Another critical challenge lies in distinguishing the effects of extracellular microbes from those of intracellular bacteria that invade tumor cells. While advances in bacterial identification techniques have improved, deciphering the variations at species and subspecies levels in low-biomass microbial environments remains a significant challenge. Often, bacteria are classified only by their genus, which may overlook their inherent diversity and specific virulence factors that could contribute to lung cancer progression. Moving from correlational studies and predictions to experimentally determining causality and molecular mechanisms is another key obstacle. For instance, contradictory findings regarding *Fusobacterium nucleatum* in colorectal cancer suggest that not all bacterial strains are capable of stable colonization or cancer promotion (160). This highlights the need for comprehensive evaluations using multiple experimental platforms to conclusively understand the causative roles of intratumoral microbes in cancer. In the context of lung cancer, further research is required to elucidate the mechanisms by which specific microbial communities contribute to lung carcinogenesis and metastasis. Looking forward, research will also need to address the complex interactions between bacteria and other microorganisms, such as viruses, fungi, and eukaryotic microbes, within the tumor microenvironment. Understanding these trans-kingdom interactions will be crucial for fully comprehending the role of the microbiome in cancer. Transitioning from cellular models and animal systems to clinical applications presents a complex challenge, but it also offers immense potential for developing novel diagnostic tools and therapeutic strategies. In the case of lung cancer, this could include microbiome-targeted therapies aimed at modulating microbial communities to enhance treatment efficacy and improve patient outcomes (**Figure 7**).

The growing understanding of the microbiome’s components has significantly advanced our knowledge of the immunological interactions within the body. Microorganisms such as bacteria, phages, and fungi interact directly with both innate and adaptive immune systems, regulating immune responses through their metabolites. The study of intratumoral microbiota has emerged as a promising area of cancer research, offering fresh insights into the complexity of cancer biology and opening new possibilities for diagnosis and treatment (161). The TMME plays a multifaceted role in shaping the tumor’s immune landscape, which presents an opportunity to enhance the efficacy of immunotherapy. The identification and characterization of intratumoral microbiota are paving the way for innovative diagnostic tools and therapeutic strategies. The potential to use microbial signatures as biomarkers for cancer diagnosis and prognosis is particularly compelling. This approach could lead to more personalized and effective treatments tailored to the individual’s tumor microbiome, particularly in lung cancer, where recent studies have revealed how specific bacterial populations in the tumor microenvironment can influence tumor progression, immune evasion, and response to treatment (162). Similarly, targeting the microbiota as a therapeutic strategy holds great promise, either as a standalone treatment or in combination with conventional therapies like chemotherapy, targeted therapy, or immunotherapy. In lung cancer, for instance, studies have suggested that the presence of specific bacteria could either enhance or hinder the effectiveness of immune checkpoint inhibitors. Thus, microbiome-targeted therapies could potentially improve the clinical outcomes of such treatments. This review has emphasized the strides made in understanding the presence, diversity, and functional impact of microbial communities within cancer ecosystems, including lung cancer. These findings challenge the traditional view of cancer as solely a disease of human cells and reveal a dynamic interaction between the host, the tumor, and the microorganisms.

Despite significant progress, translating microbiome discoveries into clinical applications faces multifaceted challenges. Technical limitations persist in characterizing the tumor microenvironment’s complexity, particularly in low-biomass settings like lung cancer where contamination risks (environmental/reagent-derived DNA), batch effects from procedural variability, and inadequate signal-to-noise ratios threaten data validity. Standardization deficits in sample collection, DNA extraction, and sequencing protocols further compromise reproducibility. These methodological constraints necessitate: (1) advanced technologies for reliable microbial detection, (2) personalized treatment approaches to address interpatient microbial heterogeneity, and (3) rigorous ethical frameworks for microbiome modulation. Concerted efforts to overcome these barriers are critical for advancing translational applications. In particular, the impact of “kitome”—microbial DNA contamination introduced by laboratory reagents—poses a significant obstacle in low-biomass studies. Reagent-derived signals can obscure true biological variation and lead to erroneous interpretations of microbial presence. To mitigate this, the implementation of rigorous negative controls at every experimental stage is essential. Furthermore, cross-laboratory validation using standardized protocols can enhance data reliability and facilitate meta-analyses across studies. These quality control measures are indispensable for distinguishing genuine tumor-associated microbial signals from experimental artifacts and ensuring the reproducibility of microbiome research in oncology. To move beyond observational associations and establish causality, the use of experimental models is indispensable. Tools such as germ-free mice, antibiotic-treated models, and tumor-derived organoids provide controlled environments for investigating how specific microbes influence tumor progression and therapeutic response. These systems enable mechanistic dissection of host-microbe interactions and allow researchers to validate the functional roles of candidate microbial species identified through sequencing studies. In the context of lung cancer, where the microbial biomass is particularly low and confounding factors are numerous, such experimental models are essential for distinguishing true microbial contributors from correlational bystanders. Incorporating these approaches will be critical for translating microbiome findings into actionable clinical interventions.

While multi-omics approaches have significantly advanced our understanding of microbial signatures associated with tumors, they remain largely correlative in nature. To unravel the causal and mechanistic roles of microbiota in tumor biology, functional studies are indispensable. These include *in vitro* assays, microbial metabolite profiling, gene knockdown experiments, and *in vivo* validation using germ-free or gnotobiotic models. Such investigations are crucial for dissecting how microbial-derived factors modulate oncogenic signaling pathways, immune responses, and therapeutic resistance. Integrating functional evidence with multi-omics data will provide a more comprehensive and actionable understanding of host–microbiota interactions in cancer. The spatial heterogeneity of the intratumoral microbiome introduces a critical dimension to tumor biology that remains underexplored (163–165). Emerging evidence indicates that microbial communities exhibit considerable variation not only between patients but also across distinct regions within the same tumor (166). Such heterogeneity may profoundly affect localized immune modulation, therapeutic response, and microbial-driven oncogenic pathways. To accurately delineate these spatial patterns, future studies should incorporate region-specific sampling and spatially resolved multi-omics or imaging approaches. Elucidating this intratumoral variability is essential for refining microbiome-based diagnostics and tailoring precise, region-informed therapeutic interventions. To elucidate the spatial dynamics and functional relevance of the intratumoral microbiome, advanced technologies such as spatial transcriptomics and single-cell sequencing should be employed (163, 167, 168). These approaches enable high-resolution mapping of microbial localization within tumor tissues and facilitate the identification of direct or indirect interactions between microbes and specific cell populations, including immune cells, cancer cells, and stromal components. By integrating spatial and single-cell data, researchers can gain mechanistic insights into how the microbiota modulates tumor biology, immune responses, and therapeutic efficacy at a cellular and molecular level. Such tools are essential for moving beyond descriptive associations toward a more comprehensive understanding of microbe-host interactions in the tumor microenvironment. The realization that cancer is not only driven by cellular factors but also by microbial elements represents a paradigm shift in our understanding of oncogenesis. This new perspective enriches our comprehension of cancer biology and propels the development of potentially transformative therapeutic interventions. As we continue to explore the role of intratumoral microbiota, it is becoming increasingly clear that the microbial inhabitants of tumors are far from passive bystanders—they play an integral role in shaping cancer progression and response to treatment.

Beyond bacteria, other microbial kingdoms such as fungi and viruses have emerged as influential constituents of the tumor microenvironment (20, 65, 169–171). Recent studies highlight the significance of trans-kingdom interactions—dynamic relationships among bacteria, fungi, and viruses—in shaping tumor immunity and disease progression (172). For instance, fungal dysbiosis has been associated with pro-tumorigenic inflammation, while certain oncogenic viruses can modulate immune evasion and treatment resistance (114). These microbial communities do not exist in isolation; rather, their synergistic or antagonistic interactions may orchestrate host immune responses, metabolic reprogramming, and even therapeutic efficacy. Therefore, comprehensive multi-kingdom analyses are essential to fully understand the ecological and functional dynamics of the tumor-associated microbiota. Integrating microbial profiling into clinical workflows holds considerable promise for advancing precision oncology. By identifying patient-specific microbial signatures, clinicians could stratify patients based on predicted responses to immunotherapy, select the most appropriate treatment regimens, and monitor microbial dynamics as a biomarker for therapeutic efficacy or resistance. For example, the enrichment of certain bacterial taxa may serve as a predictive biomarker for response to immune checkpoint inhibitors. However, translating these insights into actionable clinical tools presents multiple challenges. These include the need for rapid, cost-effective, and standardized sequencing technologies; validation of microbial biomarkers across diverse patient populations; and the integration of microbiome data with existing clinical and multi-omics datasets. Furthermore, ethical, regulatory, and logistical hurdles must be addressed before personalized microbiome-based interventions can be routinely implemented in clinical practice.

While modulating the intratumoral microbiome holds therapeutic promise, it also raises critical ethical, safety, and regulatory challenges. Ethically, informed consent must explicitly address potential long-term perturbations of the host microbiome and off-target effects. From a safety perspective, live microbial therapeutics require rigorous preclinical evaluation to exclude risks such as horizontal gene transfer, systemic dissemination, and immunopathology. Regulatory frameworks for Live Biotherapeutic Products (LBPs) demand adherence to Good Manufacturing Practice (GMP) standards, comprehensive quality control assays, and phased clinical trials to demonstrate both safety and efficacy. Harmonizing these requirements across jurisdictions will be essential for the responsible translation of microbiome-based interventions into oncology practice. To capture dynamic host–microbiota interactions over time, longitudinal cohort studies are indispensable. By serially sampling tumor tissues, blood, or non-invasive specimens (e.g., sputum, stool) before, during, and after treatment, researchers can delineate temporal shifts in microbial composition and function. Such designs enable the identification of early microbial signatures predictive of therapeutic response or relapse and facilitate causal inference through time-series analyses. Moreover, integrating longitudinal microbiome data with clinical outcomes and multi-omics layers will accelerate the discovery of robust, time-resolved biomarkers and inform adaptive intervention strategies tailored to individual disease trajectories. To translate preclinical insights into patient benefit, randomized interventional trials targeting the tumor microbiome are imperative. Strategies such as adjunctive probiotics, selective antibiotic regimens, and fecal microbiota transplantation (FMT) should be evaluated for their capacity to modulate intratumoral and systemic microbial communities, enhance treatment efficacy, and maintain safety. Early-phase studies must incorporate rigorous endpoints, including changes in microbial composition, immune correlates, and clinical outcomes, alongside comprehensive safety monitoring for off-target effects and dysbiosis. Designing such trials in lung cancer cohorts will establish proof-of-concept for microbiome modulation, inform optimal dosing and delivery methods, and pave the way for larger efficacy studies.

## Conclusions

The exploration of the intratumoral microbiome in lung cancer has unveiled its profound influence on tumorigenesis, immune modulation, therapeutic response, and metastasis. This review highlights the dynamic interplay between microbial communities (bacteria, fungi, and viruses) and the tumor microenvironment, emphasizing their roles in shaping immune evasion, metabolic reprogramming, and therapeutic resistance. Key findings underscore the potential of microbial signatures as diagnostic and prognostic biomarkers, while also pointing to novel microbiome-based therapeutic strategies, such as modulating microbial composition to enhance immunotherapy efficacy or overcome drug resistance.

Despite significant advancements, challenges remain, including technical limitations in characterizing low-biomass microbiomes, the need for standardized protocols, and the imperative to establish causal relationships beyond correlative observations. Future research should focus on integrating multi-omics approaches, spatial profiling, and preclinical models to elucidate mechanistic insights and translate findings into clinical applications. By harnessing the intratumoral microbiome’s potential, we may revolutionize precision oncology, offering innovative tools and therapies to improve lung cancer outcomes. This paradigm shift underscores the importance of interdisciplinary collaboration to fully unlock the microbiome’s role in cancer biology and treatment.
